# Investigation of Step-Stress Accelerated Degradation Test Strategy for Ultraviolet Light Emitting Diodes

**DOI:** 10.3390/ma12193119

**Published:** 2019-09-25

**Authors:** Banglong Liang, Zili Wang, Cheng Qian, Yi Ren, Bo Sun, Dezhen Yang, Zhou Jing, Jiajie Fan

**Affiliations:** 1 School of Reliability and Systems Engineering, Beihang University; Beijing 100191, China; 2 College of Mechanical and Electrical Engineering, Hohai University, Changzhou 213022, China

**Keywords:** UV-LED, step-stress accelerated tests, degradation rate, failure mechanism consistency, test strategy

## Abstract

III-nitride-based ultraviolet light emitting diode (UV LED) has numerous attractive applications in air and water purification, UV photolithography, and in situ activation of drugs through optical stimulus, solid state lighting, polymer curing, and laser surgery. However, the unclear failure mechanisms and uncertainty reliability have limited its application. Therefore, a design of an appropriate reliability test plan for UV LEDs has become extremely urgent. Compared to traditional reliability tests recommended in LED lighting industry, the step-stress accelerated degradation test (SSADT) is more cost-effective and time-effective. This paper compares three SSADT testing plans with temperature and driving currents as stepwise increasing loads to determine an appropriate test strategy for UV LEDs. The study shows that: (1) the failure mechanisms among different SSADT tests seem to be very different, since the driving current determines the failure mechanisms of UV LEDs more sensitively, and (2) the stepped temperature accelerated degradation test with an appropriate current is recommended for UV LEDs.

## 1. Introduction

Ultraviolet (UV) light has been widely used in numerous applications including wastewater treatment and reuse, counterfeit detection, lighting, fluoro-sensing, and more [1,2,3,4]. As the emitter of ultraviolet radiation, the ultraviolet light emitting diodes (UV LEDs) exhibit significant advantages compared to the traditional counterparts due to their high reliability and environmental friendliness, especially in UV-A (315–400 nm) scenarios [4]. However, in practice, many UV LEDs exhibit extremely short lifetimes due to inner defects existing in these packages, while the others work very well [5,6,7]. The problem caused by such an uncertainty in the operation lifetimes has become a serious obstacle to the application of UV LED packages, and, therefore, needs to be tackled by using typical test techniques related to reliability assessments and lifetime predictions [8,9,10,11].

Currently, the IES-TM-21 (Projecting Long Term Lumen Maintenance of LED Light Sources) standard and its extension version IES-TM-28 (Projecting Long-Term Luminous Flux Maintenance of LED Lamps and Luminaires) recommended by the Illuminating Engineering Society of North America (IESNA) are the most commonly used lifetime prediction methods for LEDs. They propose a statistical regression method to predict the long-term lumen maintenance of an LED light source. The collected lumen maintenance data is based on 6000 h (or more) of testing following the IES-LM-80 standard. In general, the luminous depreciation test requires a minimum of 6000 h testing period, which is comparable, or even longer than the period for developing a new generation of LED lighting products. This necessitates an urgent study on the development of accelerated degradation tests (ADTs) for LED packages and systems. At the package level, a number of statistic-based approaches are discussed for lifetime estimation of LEDs with the consideration of a random variation in the experimental results of ADTs. At the product level, a temperature-driven accelerated test method is developed to reduce the testing time within 2000 h. In addition, this method serves as a quick and fast pass/fail luminous depreciation qualification test for LED lamps and luminaires with an expected lifetime of 25,000 h.

The constant-stress accelerated degradation test (CSADT) and step-stress accelerated degradation test (SSADT) are the two main kinds of tests frequently observed in reliability research studies [12,13]. For UV LEDs, the former is developed based on the assumption that their optical radiation power degradations can be effectively accelerated by increasing driving currents (including both direct currents and impulse currents) and environmental temperatures [14,15,16,17,18,19]. However, the test duration of a CSADT test is highly dependent on the expected lifetime of devices under the test (DUTs) [20]. Moreover, in order to calculate the acceleration factor, at least two CSADT tests with different stress levels have to be performed. Therefore, for device under tests (DUTs) with a long-expected lifetime, the CSADT test period and cost are too much of a burden. To solve this problem, the SSADT technique can be regarded as one effective alternatives, since it combines the effects of multiple stress levels in a single test. The SSADT test has been already successfully employed in several electronics and LEDs for lifetime predictions such as by He [21], Cai [22,23], Qian [24], Wang [25], Benavides [26], Tseng [27], Hu [28], etc. However, applications of using SSADT in UV LEDs are rarely found in literature.

This paper investigates the test strategy of SSADT on the reliability assessment for UV LEDs. The remainder of the paper is organized as follows: Section 2 describes the details of the DUT samples and experiment designs with four different test conditions (including three SSADT tests and one CSADT test). Section 3 explains the theory and models used to calculate the aging degradation rates of the UV LEDs’ radiation power maintenance and analyze the aging temperature and current effects. Next, the comparison and discussion on the experimental results and model predictions are performed in Section 4 to figure out the best SSADT condition for UV LEDs. Lastly, the concluding remarks of this study are summarized in Section 5.

## 2. Test Samples and Experiments

**Figure 1:**
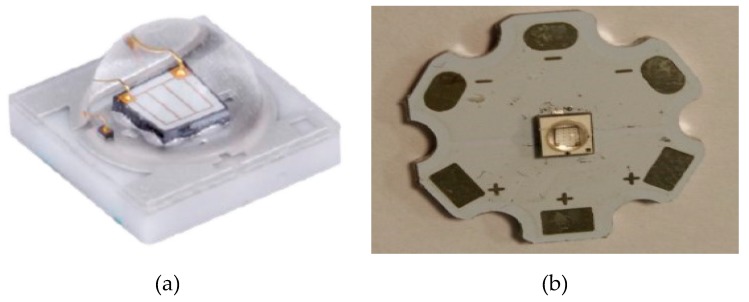
Illustrations of the test sample (**a**): UV-A LED package, and (**b**) UV-A LED mounted on an MCPCB (Metal Core Printed Circuit Board).

The UV LED sample tested in this study is produced by a commercial 3535 type UV-A LED from the Lattice Power Corporation (Changzhou, Jiangsu Province, China), shown in Figure 1a, mounted on a metal core print circuit board (MCPCB) via a eutectic die bonding process, as shown in Figure 1b. The peak emission wavelength of the test sample is between 365 nm and 375 nm, and the rated driving current is 350 mA.

**Figure 2:**
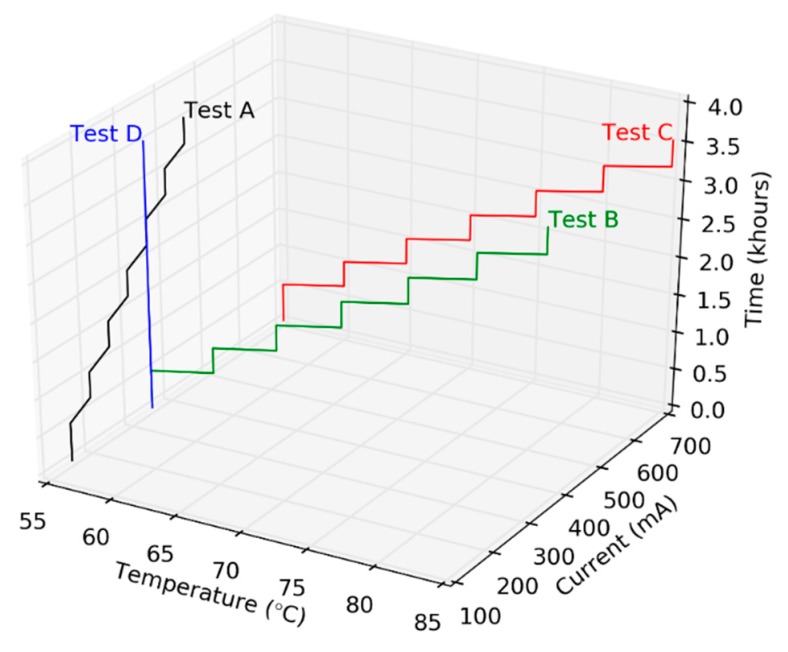
The testing profiles of four pre-assigned accelerated degradation tests.

Since the rated driving current of the selected test sample is 350 mA, we chose 700 mA, which is twice its rated current, as the accelerating condition for the test sample. The stepped testing period in SSADT tests is determined based on our previous experience on the tests for LED products [24]. The lower and upper bounds of temperatures in the temperature based SSADT tests, i.e., 55 °C and 85 °C respectively, are selected according to the current LED performance and reliability test standards, such as IES-LM-80 and IES-TM-21 [29,30]. Temperatures lower than 55 °C are not used as environmental temperatures in order to reduce the test period. On the other hand, temperatures higher than 85 °C are also not used to avoid the occurrence of side failures caused by the self-heating effect of UV LED chips. Under the guidance of the above parameters, four types of accelerated degradations tests are performed in this study. Figure 2 shows the testing profiles of these tests, in which Tests A, B, and C are SSADT tests whereas Test D is a CSADT test. Details of the tests conditions are given as follows: (1) Test A is performed under a constant temperature of 55 °C and a stepwise current from 150 mA to 450 mA with an increment of 50 mA in every 504 h. (2) Test B is performed under a stepwise temperature from 55 °C to 85 °C with an increment of 5 °C in every 504 h and a constant current of 350 mA. (3) Test C is performed under a stepwise temperature from 55 °C to 85 °C with an increment of 5 °C every 504 h and a constant current of 700 mA. (4) Test D is performed under 55 °C and 350 mA, which are exactly the maximum operation temperature and rated driving current, respectively.

In total, 15 samples are tested up to 3360 h in each of the above-mentioned tests. According to the multiplication rule specified in IES TM-21 standard, the expected lifetimes obtained from the extrapolation along the radiation power maintenance decay curves of the UV LEDs are limited no more than 5.5 times of the test period, which equals 18,480 h [30]. This is a long enough period as the rated lifetime for a vast majority of currently available UV LED products. Furthermore, in the SSADT tests (i.e., Tests A, B, and C), the degradation rate of the radiation power maintenances is supposed to vary with the increase of stress levels. Therefore, in order to acquire an accurate degradation rate in each stress level, at least four radiation power values are needed over the period of 504 h by using the linear curve fitting method. The radiation powers are measured every 168 h during the entire SSADT test. In addition, for comparison purposes, the measurement interval of the radiation powers is set as 168 h in the CSADT test (i.e., Test D) as well.

## 3. Theory and Modeling

Similar to the LEDs used in visible lighting, the degradation of radiation power maintenance of UV LEDs is assumed to follow an exponential time-dependent relationship, which can be described by Equation (1) [30].

(1) P=βexp(−αt)

where *P* indicates the radiation power maintenance defined as the ratio between the radiation power at a time *t* and its initial value, *α* is the degradation rate used for characterizing the degree of radiation power degradation, and *β* is the pre-factor. Ideally, the *β* parameter should equal to 1, which is the initial radiation power maintenance at time zero. However, in practice, it is extracted with a slight offset due to the factors such as the measurement error and lateral failure mechanisms.

For an SSADT test, by assuming that the damages induced from different stepwise loading conditions are not correlated with each other, Equation (1) can be generalized into consecutive exponential time-dependent curve segments, as shown in Equation (2).

(2) $$P_{i}=\{\begin{array}{ccc} \beta_{1}exp\left(-\alpha_{1}t\right), & & 0\leq t\leq t_{1}\\ & \ldots & \\ \beta_{i}exp\left(-\alpha_{i}t\right), & & t_{i-1}\leq t\leq t_{i}\\ & \ldots & \\ \beta_{n}exp\left(-\alpha_{n}t\right), & & t_{n-1}\leq t\leq t_{n} \end{array}$$

where *n* is the total numbers of stepped stress levels, and *α_i_* and *β_i_* are the degradation rate and pre-factor extracted under the *i*^th^ stress level.

Certainly, the degradation rate of radiation power maintenances of UV LEDs will be affected by both thermal and electrical stresses applied on them. Such effects can be described by a general function given below [20].

(3) $$\alpha =exp\left\{ln\left(\gamma_{0}\right)-\frac{E_{a}}{kT}+\gamma_{2}ln\left(I\right)+\frac{\gamma_{3}ln\left(I\right)}{kT}\right\}$$

where *I* and *T* indicate the driving current and temperature, *γ*_0_, *γ*_2_, and *γ*_3_ are empirical constants, *E_a_* is the activity energy, and *k* is the Boltzmann constant. As indicated in Equation (3), it considers the effects of temperature, current, and temperature-current interaction in the degradation rate.

It can be seen from Figure 2, the stepwise increments of temperature and current are not mutually exerted during the SSADT tests. For those tests with stepwisely increased temperatures, Equation (3) can be simplified as:

(4) $$\alpha =exp\left\{a+\frac{b}{T}\right\}$$

However, for those tests with step-wisely increased currents, Equation (3) can be simplified as:

(5) α=exp{a+bln(I)}

In Equation (4) or Equation (5), *a* and *b* are fitting parameters and the degradation rates (i.e., *α* parameter) can be extracted from their experimental results of Tests A, B, and C. The extracted *α* parameters from the different tests are compared to determinate a proper SSADT test plan for the UV-A LEDs used in this study.

## 4. Results and Discussions

**Figure 3:**
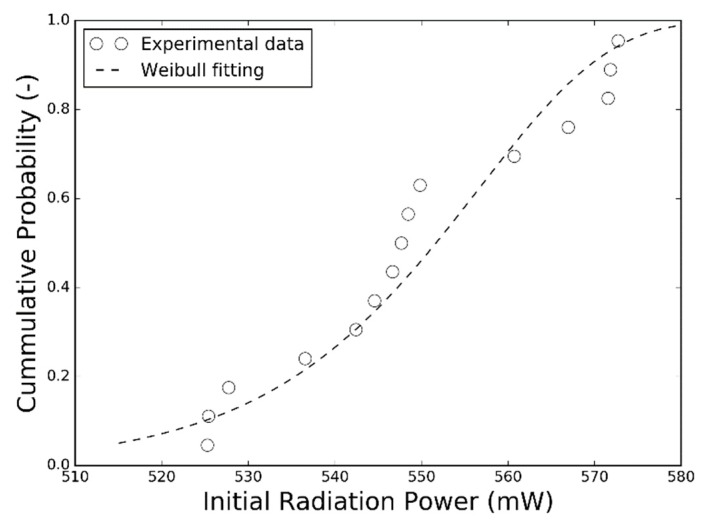
Initial radiation power measurements of a batch of samples.

**Figure 4:**
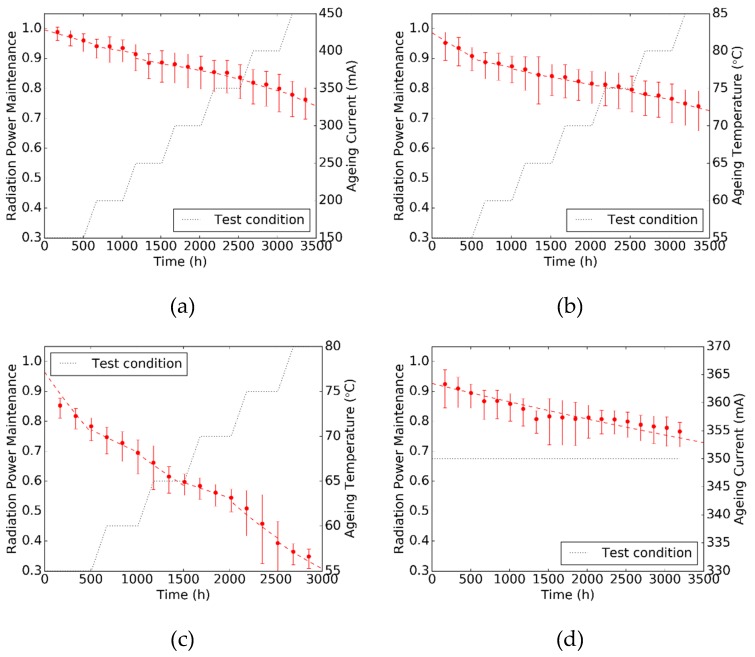
Experimental results of the radiation power maintenances from four different tests. (**a**): Test A; (**b**): Test B; (**c**): Test C; (**d**): Test D.

Initial radiation powers measured from a batch of samples before the SSADT test are plotted in Figure 3 to reveal the statistical character of the test samples. These initial radiation power measurements vary between 520 mW to 580 mW, and can be considered to follow a two-parameter Weibull distribution. The radiation power maintenances, calculated by the ratio in between the real-time radiation power and its initial value, of test samples from Tests A, B, C, and D are then plotted in the sub-graphs of Figure 4, in which the left and right ordinates represent the radiation power maintenance and stepped test condition, respectively. By assuming the radiation power maintenance measurements at each time point, and following the two-parameter Weibull distribution, their median values and error bars are calculated and plotted by red dots and vertical red bars, respectively. The entire degradation curves of radiation power maintenances in Tests A, B, and C is formed by connecting a couple of degradation curve segments extracted sequentially under the stepwise increasing load conditions. It can be seen that the degradation trends of these entire degradation curves are observed nearly continuously even though the continuity is not taken into account in Equation (2). Based on these radiation power maintenance measurements, the degradation curves are extracted by Equation (2) using the least square method, and plotted in red dashed lines in Figure 4 as well. It can be seen that the median values of the radiation power maintenances are in good agreement with the degradation curves in all of the four accelerated degradation tests. This proves that Equation (2) is adequately accurate to describe the degradation trends of radiation power maintenances of the test samples under different test conditions.

**Figure 5:**
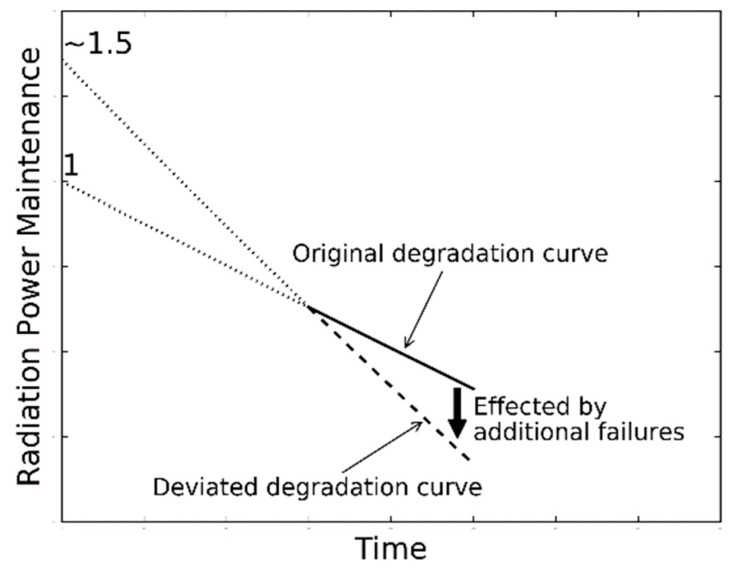
Influence of the additional failures on the pre-factor of the degradation model.

**Figure 6:**
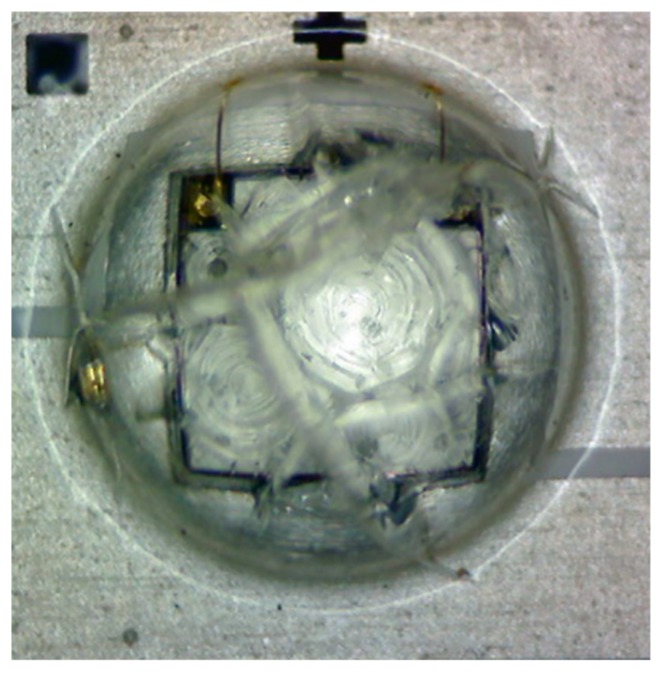
Silicone encapsulant splitting observed in a sample after Test C.

Based on the experimental results in Figure 4, the degradation rate *α* and pre-factor *β* of the test samples under each pair of temperature and current stress level in Tests A, B, C, and D are extracted and shown in Table 1. For those samples in Test C, the extracted *β* parameters are increased to about 1.5 after the aging temperature goes up to 75 °C, which implies that additional failures occur as illustrated in Figure 5. This coincides with the evidence that the silicone encapsulant of the test sample is seriously cracked at the end of Test C, as shown in Figure 6. Such a failure is possibly caused by overloaded thermal stress in the body of silicone encapsulant, and can be observed in all samples after Test C. Furthermore, the *β* parameter extracted from Test A shows an increasing trend when the driving current increases to 450 mA. This implies 450 mA could be the highest current for the test samples to conduct an SSADT. If the current is further elevated, additional failures such as encapsulant cracks might happen in those samples. Nevertheless, within the whole period of Tests A, B, and the first half of Test C, it is believed that the failure mechanism remains consistent, since the extracted *β* parameters are not significantly varied.

**Figure 7:**
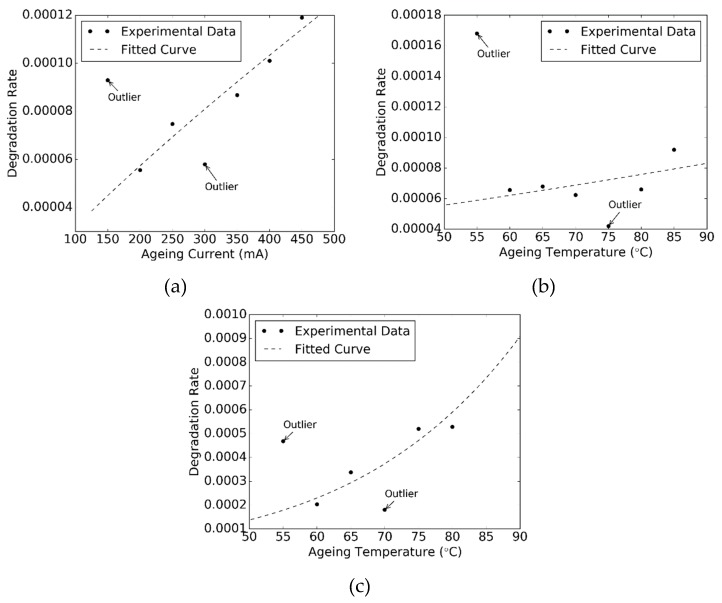
Extracted degradation rates from the SSADT tests and the fitted curves to Equation (4) and Equation (5), respectively. (**a**): Test A; (**b**): Test B; (**c**): Test C.

Figure 7 plots the relationship in between extracted *α* parameters and the aging currents/temperatures obtained from Tests A, B, and C and their fitted curves to Equation (4)/Equation (5), respectively. As shown in Figure 7, the outliers, which significantly deviated from the trend, are described by Equation (4)/Equation (5) and detected with studentized residuals by the following steps. These outliers are eliminated in the curve fitting process.

1. Transform Equation (4) and Equation (5) to a linear relationship, which is shown in the formula below.

(6) $$y=C_{1}x+C_{2}$$

where *C*_1_ and *C*_2_ are model constants, *y* indicates ln(*α*), and *x* indicates the 1/T or ln(I) depending on the certain equation.

2. Calculate the studentized residual of each y by using Equation (7).

(7) $$r_{i}=\frac{e_{i}}{\sigma \sqrt{\left(1-h_{ii}\right)}}$$

where *e*, *σ*, and *h_ii_* are calculated by the following equations.

$$e_{i}=\mathrm{abs}\left(y-y^{\prime }\right),\;\sigma =\sqrt{\frac{\sum_{i=1}^{n}e_{i}^{2}}{n-2}},\;h_{ii}=\frac{1}{n}+\frac{{\left(x_{i}-x_{avg}\right)}^{2}}{\sum_{j=1}^{n}{\left(x_{j}-x_{avg}\right)}^{2}}$$

where *y′* indicates the estimate of *y*, *n* indicates the sample size, and *x_avg_* is the mean of all *x* values.

3. Find out the *y* values with studentized residuals higher than a threshold (which equals to 2 in this study), and indicate them as the outliers.

From Figure 7, it can be seen that the fitted curves to *α* parameters agree with the experimental values very well in all the SSADT tests (i.e., Tests A, B, and C). The resulting fitting parameters of these curves are given in Table 2. It is found that Tests B and C result in completely different fitting parameters of Equation (5). This can be attributed to the fact that different failure mechanisms might arise from the very beginning because of the large discrepancy in driving currents between these two SSADT tests.

With the extracted parameters shown in Table 2, the degradation rates *α* of radiation power maintenances of the samples aged at 55 °C and 350 mA are predicted by using Equation (4)/Equation (5), and displayed in Table 3. Moreover, these predicted *α* parameters are compared with the experimental *α* parameter extracted directly from the test results of Test D (i.e., 6.857 × 10^−5^). The error in Table 3 indicates the offsets in between the prediction and experimental *α* values. Among the three SSADT tests, the best prediction of the *α* parameter is given by Test B with an error of −14.1%, which is followed by Test A with an error of 34.4%. A common feature is that the driving current applied on these two SSADT tests is moderate and close to that applied on Test D. In contrast, with an overdrive current as high as 700 mA, Test C gives a prediction of *α* parameter with a huge error. Such a large positive bias is attributed to the additional failures (such as encapsulant cracks shown in Figure 6) observed during Test D. These additional failures drastically reduce the external quantum efficiency of the UV LEDs, and, therefore, lead to an exaggerated degradation on radiation power maintenances. Therefore, a conclusion can be drawn that a good SSADT test strategy for UV-A LEDs should consist of stepwise increasing temperatures with an appropriate driving current.

## 5. Conclusions

In this paper, three SSADT tests by considering both effects of aging temperature and driving current and a CSADT test are performed to determine a proper test strategy for UV LEDs. The degradation rates of UV LEDs aged under different conditions are calculated by fitting the proposed models and their failure mechanisms are also analyzed. The results indicate that: (1) the failure mechanism remains unchanged all through each SSADT test except for the end of over-driving the current test in which a serious rupture is observed on silicone encapsulant among all test samples. (2) The degradation rates of test samples aged at 55 °C and 350 mA are predicted from SSADT tests and, compared to the CSADT test result, in which Test B provides the best prediction accuracy. (3) Since we found the driving current plays a more determinative role in the SSADT test planning, it is recommended that the SSADT test for UV LEDs should be carried out by stepwise increasing temperatures with an appropriate driving current. This driving current could be determined from 350 mA to 450 mA, according to this research.

## Figures and Tables

**Table 1:** Degradation model parameters extracted from all four tests.

Test ID	T (°C)	I (mA)	*α*	*β*
Test A	55	150	9.294 × 10^−5^	1.00
	55	200	5.546 × 10^−5^	0.98
	55	250	7.473 × 10^−5^	0.99
	55	300	5.791 × 10^−5^	0.96
	55	350	8.670 × 10^−5^	1.03
	55	400	1.009 × 10^−^^4^	1.07
	55	450	1.189 × 10^−^^4^	1.13
Test B	55	350	1.683 × 10^−^^4^	0.99
	60	350	6.555 × 10^−5^	0.93
	65	350	6.790 × 10^−5^	0.93
	70	350	6.228 × 10^−5^	0.92
	75	350	4.202 × 10^−5^	0.88
	80	350	6.597 × 10^−5^	0.93
	85	350	9.194 × 10^−5^	1.00
Test C	55	700	4.683 × 10^−^^4^	0.97
	60	700	2.033 × 10^−^^4^	0.86
	65	700	3.380 × 10^−^^4^	0.97
	70	700	1.804 × 10^−^^4^	0.78
	75	700	5.204 × 10^−^^4^	1.52
	80	700	5.278 × 10^−^^4^	1.50
Test D	55	350	6.857 × 10^−5^	0.93

**Table 2:** Fitting parameters of the degradation rate model obtained from different SSADT tests.

Test ID	Relevant Model	*a*	*b*
Test A	Equation (5)	−14.27	0.85
Test B	Equation (4)	−6.17	−1172.23
Test C	Equation (4)	8.24	−5532.46

**Table 3:** Prediction results of the degradation rates by the SSADT tests.

Test ID	*α*	Error
Test A	9.223 × 10^−5^	34.4%
Test B	5.886 × 10^−5^	−14.1%
Test C	1.787 × 10^−^^4^	160.9%
